# Evolution of lake microbial structure and function under climatic and anthropogenic forcings: Insights from sedimentary ancient DNA

**DOI:** 10.1016/j.isci.2026.117760

**Published:** 2026-10-03

**Authors:** Xinjie Shi, Hai Xu, Wanzhu Li, Jing Wang, Yunping Song, Meiling Yang, Baoli Wang, Cong-Qiang Liu

**Affiliations:** 1 Key Laboratory of Karst Georesources and Environment, Ministry of Education, College of Resources and Environmental Engineering, Guizhou University, Guiyang 550025, China; 2 Institute of Surface-Earth System Science, School of Earth System Science, Tianjin University, Tianjin 300072, China; 3 Tianjin Bohai Rim Coastal Earth Critical Zone National Observation and Research Station, Tianjin 300072, China

**Keywords:** anthropogenic climate change, lake, microbial community, function, centennial-scale evolution, carbon stable isotope

## Abstract

Structure and function of lake microorganisms are sensitive to environmental disturbances; yet their differential responses to climatic and anthropogenic forcings over centennial scales remain poorly understood. Here, we analyzed a sediment core from Lake Daihai in northern China, combining metagenomics and stable isotope analyses. Our results revealed that microbial community assembly was dominated by deterministic processes, whereas stochasticity increased notably after 1850, coinciding with the termination of the Little Ice Age and intensified human impacts. Notably, microbial community structure evolved at a rate approximately one order of magnitude faster than community function. This decoupling was mediated by functional redundancy associated with bacterial keystone taxa, ultimately resulting in more negative sedimentary carbon and nitrogen isotopic compositions nearly a century later. Gene-level functional redundancy further buffered ecosystem function variability across biological scales. Collectively, our findings integrate omics and geochemical approaches to advance the understanding of lake ecological evolution under complex multi-forcing regimes.

## Highlights

• Microbial community structure and function showed divergence over past 500 years
• Key bacteria taxa mediated the divergent evolution throughout the period
• Impact of stochastic processes on microbial community assembly increased after 1850
• Functional redundancy buffered ecosystem function variability

## Graphical abstract

**Figure:**
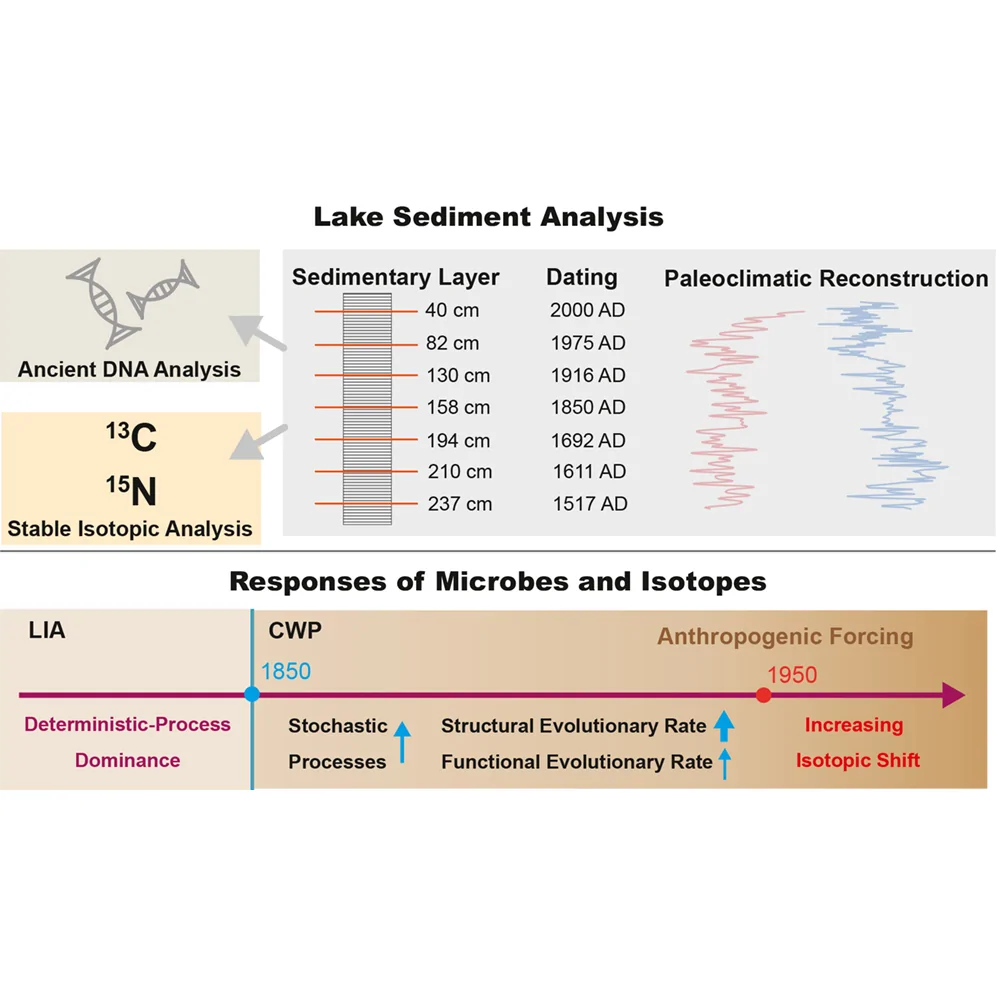

## Teaser

Environmental science; Ecology; Microbiology

## Introduction

Climate change has shaped life evolution on Earth over geological timescales, and over the past few decades, human activities have become an important driver of climate change.^1^^,^^2^ Assessing the current and future impacts of this anthropogenic climate change on ecosystems largely depends on understanding how organisms within these ecosystems respond.^3^^,^^4^^,^^5^ Although microorganisms play a critical role in modulating climate processes, they have historically received limited attention in climate change research until 2019, when scientists reached a consensus to integrate microbes into the bio-environmental feedback framework under climate change.^6^^,^^7^^,^^8^ In this context, compared with the well-documented community succession and functional dynamics of microorganisms in response to environmental changes on seasonal or decadal scales, centennial-scale microbial evolution can directly reveal their feedback loops with climate change.^9^^,^^10^^,^^11^^,^^12^ Therefore, elucidating the central role and evolutionary significance of microbes in climate change biology is essential for guiding ecological sustainability efforts.

Lakes play a crucial role in global biogeochemical cycles and provide essential ecosystem services to humanity, such as maintaining biodiversity and supplying water resources.^13^ As key drivers of nutrient biogeochemical cycling and energy transformation in lakes, microorganisms have evolved with climate change over geological timescales,^5^^,^^14^^,^^15^ and their response-feedback mechanisms are pivotal in shaping the self-regulating capacity of lake ecosystems over time as well as determining their potential to withstand future disturbances.^8^^,^^16^ Over the past several centuries, anthropogenic climate change has become an important driving force for lake evolution, affecting microbial community composition and function, and ultimately modulating the ecological resilience of lakes.^17^^,^^18^ However, in contrast to the rapid response of microbial communities to environmental changes, the impacts on microbial functions are collectively shaped by multiple processes: the physiological acclimation of existing taxa, the differential survival and reproduction of specific taxa, the dispersal and colonization of taxa from other ecosystems, and evolutionary processes driven by shifts in allele frequencies or genetic mutations and recombination within strains.^19^ Given these distinct response patterns of microbial structure and function under disturbances, we hypothesize that they will demonstrate divergent evolutionary trends over a centennial timescale.

Lake sedimentary microbiomes record temporal changes of microbial structure and function, and when combined multiple paleoclimate proxies, they can further reflect lacustrine eco-evolution under climate change.^20^^,^^21^ To verify the aforementioned hypothesis, we therefore investigated the sediments of Lake Daihai in northern China, examining the evolutionary characteristics of microbial community composition and function during the Little Ice Age (LIA) and the Current Warm Period (CWP) primarily through metagenomic and stable isotope analyses. The main aims of this study were to: (1) characterize the centennial-scale evolution of microbial structure and function in lakes; and (2) explore the eco-evolutionary pattern of microorganisms under anthropogenic climate change. This study is expected to improve our understanding of lake microbial eco-evolutionary dynamics under anthropogenic forcings on a centennial scale.

## Results

### Microbial and geochemical parameters

For microbial community composition, bacteria were the top with a relative abundance of approximately 83%, meanwhile eukaryotes were the least with a relative abundance of approximately 0.05% (Figure 1). Interestingly, the percentage of bacteria declined, whereas those of archaea, viruses, and eukaryotes increased over time, especially after 1850. For microbial functional pathways, there were six annotated pathways, of which metabolism and environmental information processing were the top two pathways with their averages of approximately 16% and 4%, respectively (Figure 1). The annotated pathways showed a clear decrease in proportion after 1850.

**Figure 1:**
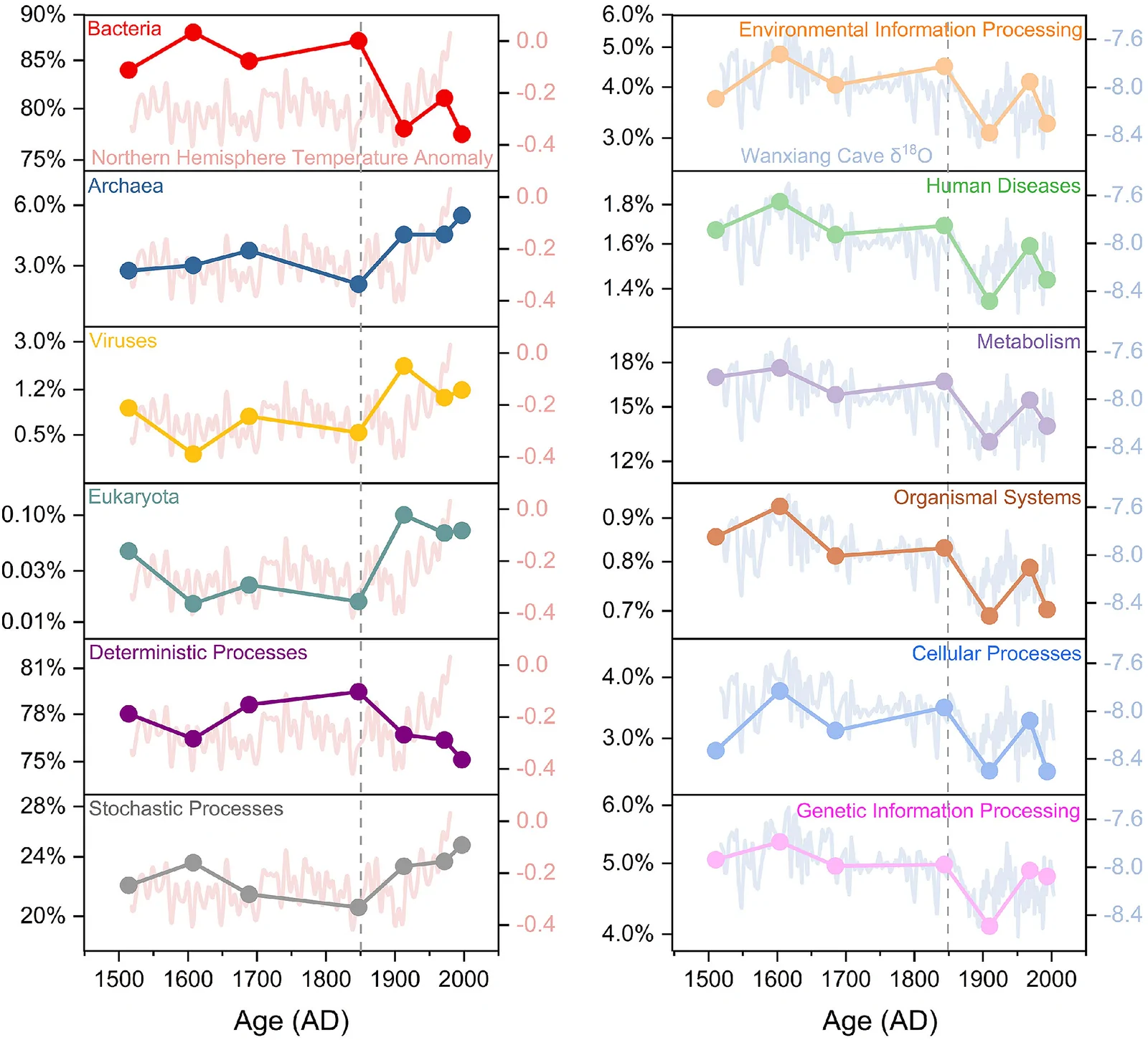
Sedimentary microbial community composition and assembly, functional pathways, and climate conditions over the past 500 years The dotted line was the line of demarcation between Little Ice Age and Current Warm Period. The stalagmite δ^18^O records from Wanxiang Cave (an indicator of EASM) and Northern Hemisphere Temperature anomaly were referred to understand the climatic conditions over the past 500 years.^22^^,^^23^

For microbial community assembly, the deterministic processes were dominant, accounting for approximately 77.0%, and declined 2.1% after 1850 (Figure 1). The positive cohesion of microorganisms was greater than the negative cohesion (an average Pos/Neg of approximately 1.26). During the LIA, the genomic GC content ranged from 53.23% to 60.41%, with an average of 55.79%, while during the CWP, the GC content ranged from 50.91% to 52.34%, with an average of 51.53%. The phylogenetic diversity (PD) and niche of microbial community were approximately 49 and 5.1, respectively. The GC value and niche decreased, while PD and the Pos/Neg ratio increased over time; their variations increased obviously after 1850 (Figure S1).

Sedimentary C/N ratios ranged from 10.6 to 11.9, with an average of 11.1.^24^ During the LIA and CWP, sedimentary δ^13^C_o_ᵣ_g_ values ranged from −25.4‰ to −23.8‰ and from −24.4‰ to −23.9‰, with averages of −24.2‰ and −24.5‰, respectively; sedimentary δ^15^N_o_ᵣ_g_ values ranged from 4.7‰ to 5.9‰ and from 2.6‰ to 5.0‰, with averages of 5.3‰ and 3.9‰, respectively. Sedimentary C/N ratios, δ^13^C_org_, and δ^15^N_org_ sharply changed from 1916 to 1975 (Figure S1). However, there was no significant difference in the aforementioned variables between LIA and CWP (*p* = 0.99, *p* = 0.57, and *p* = 0.09, respectively).

### Relationship among parameters

There was a significant temporal decay relationship for microbial community composition but not for functional pathways (Figures 2A and 2B). Dissimilarity in microbial community absolute abundance was positively correlated with sedimentary δ^13^C_org_ dissimilarity, whereas dissimilarity in functional pathway relative abundance was negatively correlated with sedimentary δ^15^N_org_ dissimilarity (Figures 2C and 2D).

**Figure 2:**
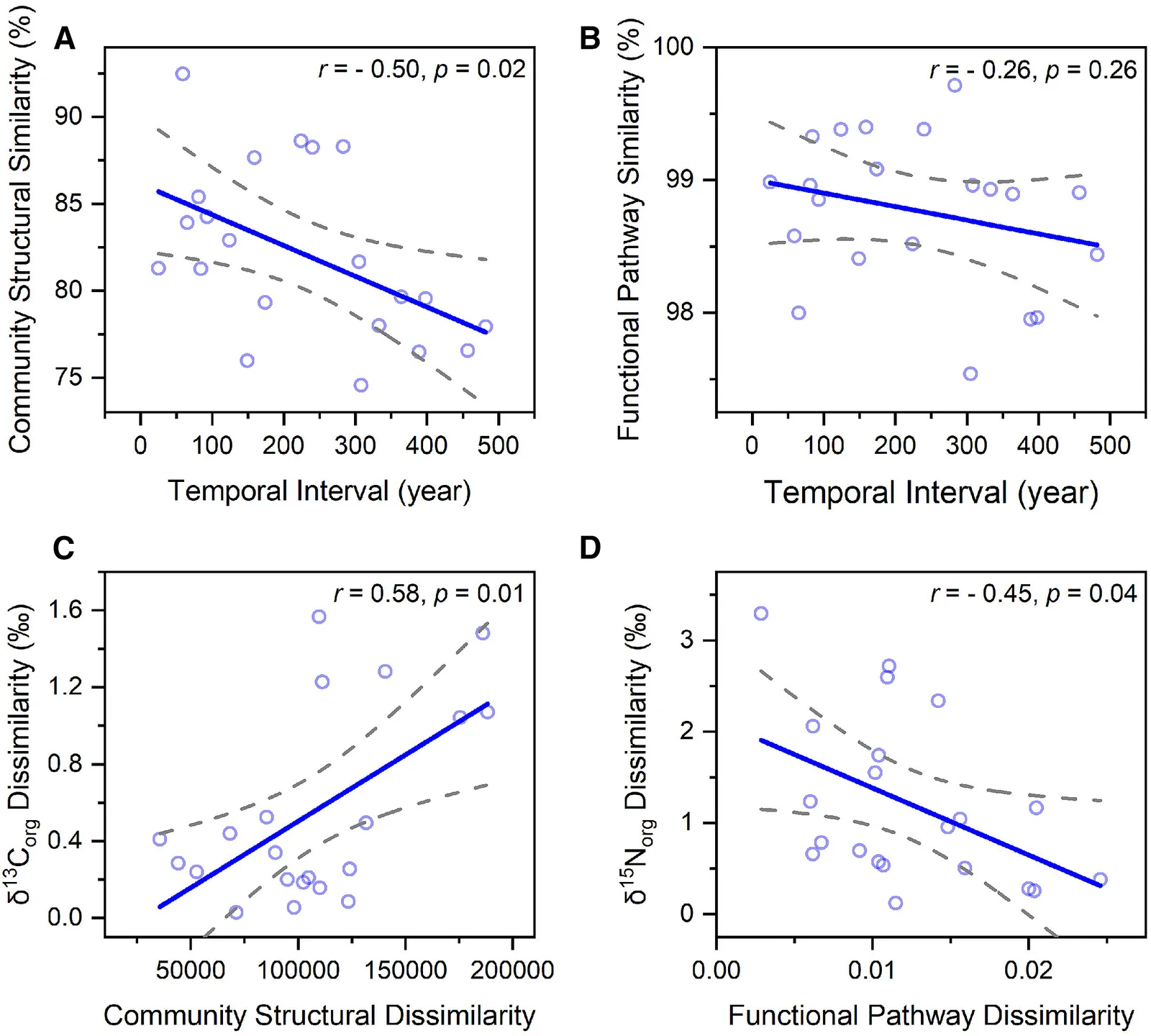
Temporal decay and isotopic dissimilarity relationships in sedimentary microbial community composition and functional pathways (A and B) Temporal decay and isotopic dissimilarity relationships in sedimentary microbial community composition and functional pathways. Temporal decay relationship for sedimentary microbial community composition and functional pathways. (C) Microbial community structural dissimilarity versus sedimentary δ^13^C_org_ dissimilarity. (D) Microbial functional pathway dissimilarity versus sedimentary δ^15^N_org_ dissimilarity. *n* = 21, n represents number of site differences. Blue line is the fitting curve. The dotted lines indicate the 95% confidence intervals.

Microbial community similarity was positively correlated with functional pathway similarity during both the LIA and the total period, and the slope during the total period was approximately 2-fold higher than during the LIA (Figure 3A). The Pos/Neg ratio and PD of microbial community significantly increased from the LIA to the CWP, while their genomic GC content and niche decreased (Figure 3B). Twenty-seven keystone taxa and twenty-nine different taxa were identified, of which seven taxa overlapped between these two groups, and their abundance significantly increased toward the contemporary period (Figure 4).

**Figure 3:**
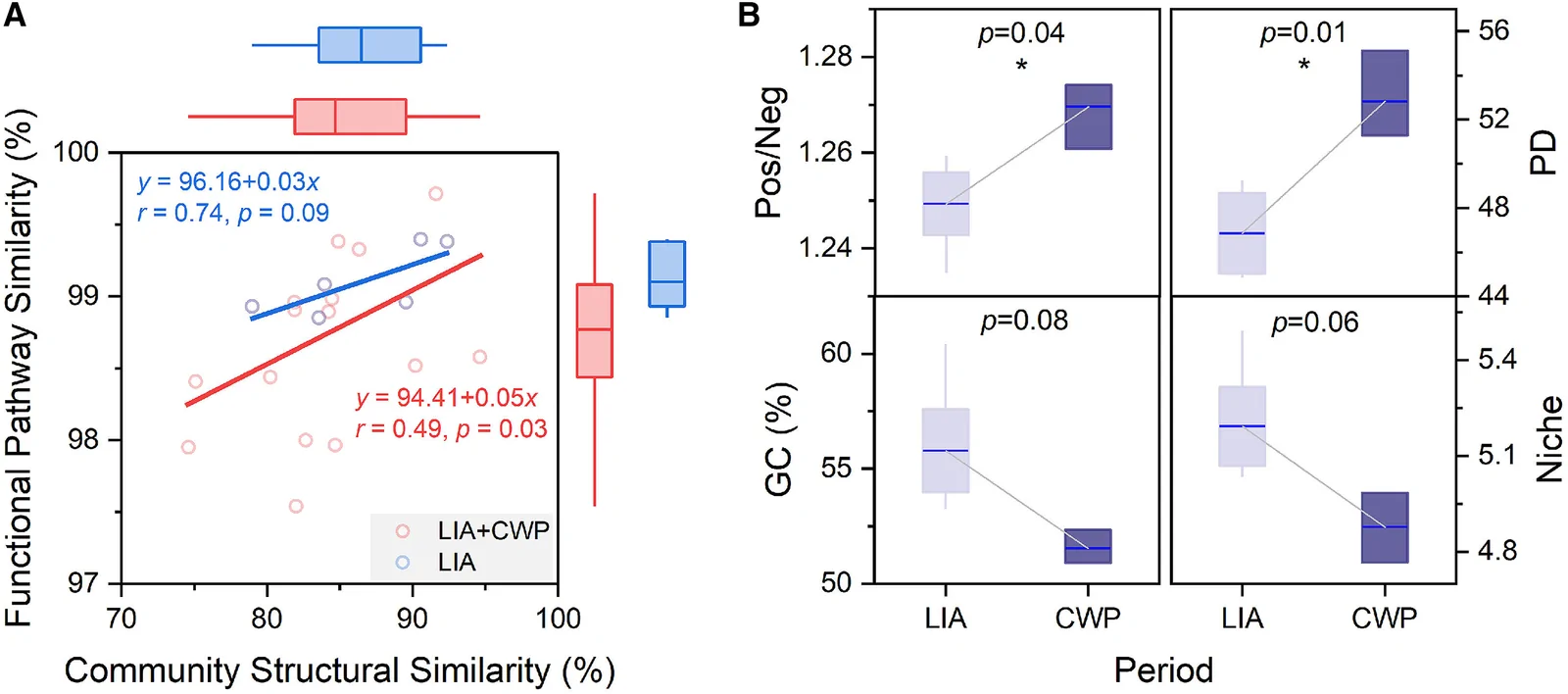
Sedimentary microbial community structure–function relationships and characteristics during the LIA and CWP (A) Sedimentary microbial community structural similarity versus functional pathway similarity during the LIA and the total period (i.e., LIA and CWP). *n* = 21, n represents number of site differences. The similarity is normalized as percentage. Boxplots indicate data ranges. (B) Boxplots of sedimentary microbial community structural and functional characteristics in LIA and CWP. Boxes represent the 25–75^th^ percentiles, whiskers are the maximum and minimum, and midline indicates the average. *n* = 7, n represents number of sites. Gray lines connect the averages between LIA and CWP, and ∗ is the significant difference (Mann-Whitney *U* test) at the level of 0.05 in (B) LIA, Little Ice Age; CWP, Current Warm Period.

**Figure 4:**
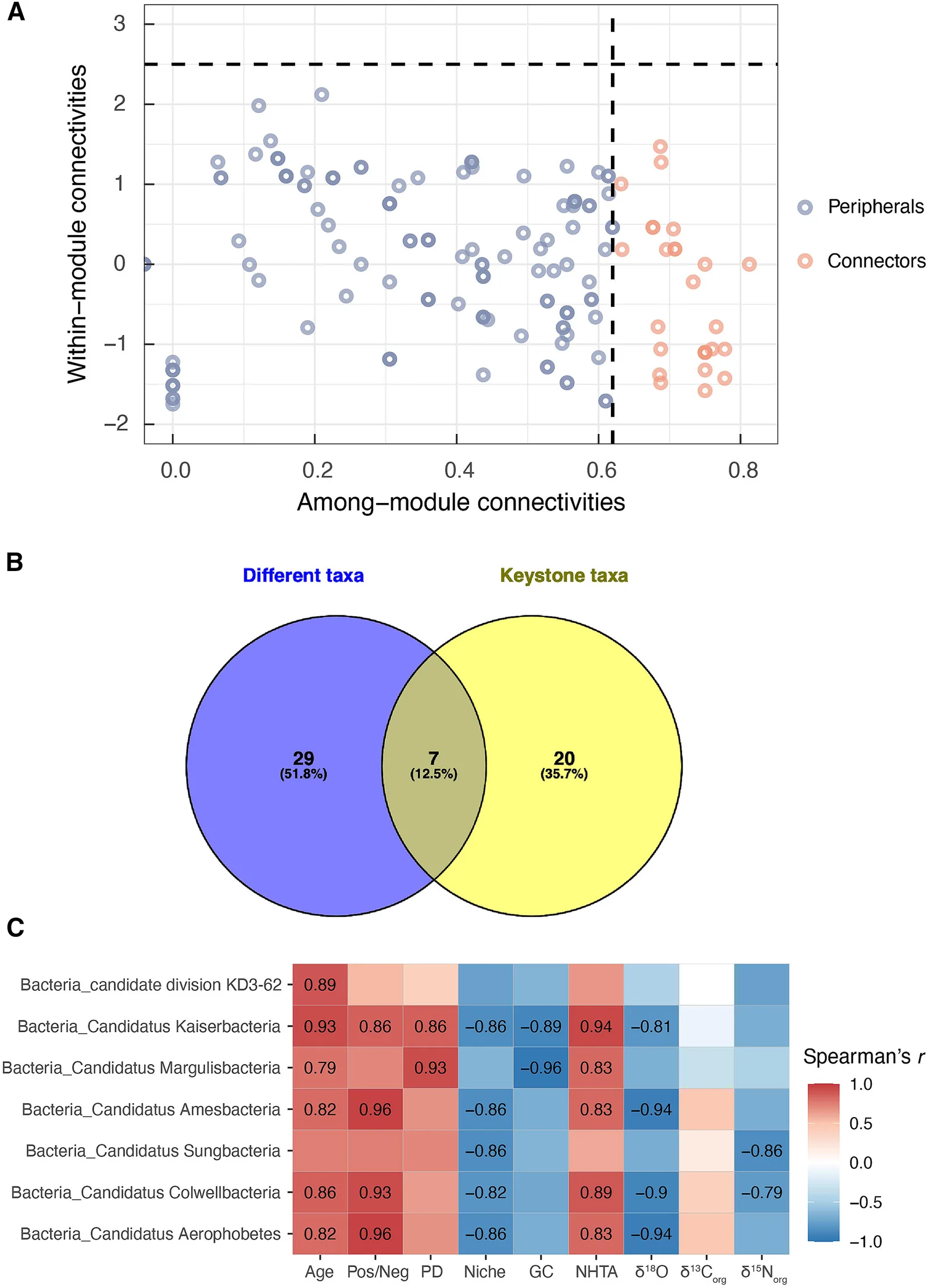
Identification of keystone taxa and their relationships with climatic and geochemical indices (A) Identification of keystone taxa using the Zi-Pi plot. Peripherals have only a few links and almost always to the nodes within their modules, whereas connectors are highly linked to several modules and act as keystone taxa in microbial co-occurrence network. (B) Venn diagram between different taxa and keystone taxa. Different taxa, their abundance having 2-fold difference at least between LIA and CWP. Seven overlapping taxa between keystone taxa and different taxa indicate the significantly different keystone taxa between LIA and CWP. (C) Spearman correlation plot between significantly different keystone taxa and climatic and geochemical indexes. The number indicates the significant correlation coefficient, significance levels: *p* < 0.05. The abbreviations are referred to the text.

## Discussion

### Evolution of lake microbial community structure and function

Molecular biological evidence indicated that microbial community structure and function have gradually evolved to exhibit new characteristics over the past approximately 500 years (Figures 2A and 2B). Although microbial community structure and function were significantly correlated in terms of their similarity, the slope was far less than 1. This suggested that microbial community structure and function underwent disproportionate evolution: the former evolved nearly 10 times faster than the latter, accompanied by strong functional redundancy (Figure 3A). The relatively smaller differentiation in microbial functional traits is likely the primary reason for the insignificant temporal decay pattern of microbial function (Figure 2B). Microbial community assembly was dominated by deterministic processes. Following 1850, with the termination of the LIA and the onset of the CWP, East Asian Summer Monsoon (EASM) significantly intensified, causing northern China from cold-dry to warm-wet conditions.^24^^,^^25^^,^^26^ This remarkable climatic transition, coupled with increasing anthropogenic disturbance (e.g., the change of land use and anthropogenic nutrient loading),^27^^,^^28^ led to the slightly increasing contribution of stochastic processes to microbial community assembly (Figure 1).^27^^,^^29^ It is probably because weak inter- and intra-specific competitive interactions among microbes during the adaptation phase created opportunities for stochastic processes such as random birth and colonization events (Figure S1).^30^ Furthermore, dispersal limitation of nutrient-sensitive taxa and increased drift in eutrophic microhabitats may raise stochastic proportion under anthropogenic nutrient loading.^27^^,^^29^ This finding aligns with observations from highly disturbed eutrophic lakes, contrasting with stronger microbial functional adaptation in ecosystems under weaker anthropogenic impacts.^18^^,^^31^

From a phylogenetic perspective, microorganisms have gradually evolved diverse taxonomic branches, including more archaeal and eukaryotic taxa (Figure S2). However, microbial functional traits are typically non-monophyletic, with multiple phylogenetic lineages possessing identical or similar functions (i.e., functional redundancy).^15^^,^^32^ This redundancy buffers the impact of species loss or succession on ecosystem functioning under environmental disturbances,^17^ thereby explaining why microbial structural evolution generally outpaces functional evolution. Upon entering the CWP, intensified anthropogenic activities have accelerated the evolution of both structure and function, particularly the latter (Figure 3A). Some stochastic processes like horizontal gene transfer could also alter phylogenetic evolution, leading to the reassignment of some functions.^32^ In any case, the increasing stochastic proportion of microbial community assembly enhanced the colonization opportunity of the taxa with unidentified functions and further promoted the functional evolution of the community. The intensity of functional redundancy during the LIA, when stochastic processes were weaker, was thus stronger than that across the studied centennial scale (Figure 3A). Therefore, the asynchronous evolution of lake microbial community structure and function over centennial scales may be the integrated outcome of deterministic selection and stochastic modification of their phylogenetic differentiation.

Keystone taxa play a key role in the response of microbial community structure and function to climate change.^33^ Interestingly, the distinct keystone taxa identified between the CWP and LIA were all annotated as bacteria and generally responded to the variable temperature and rainfall (Figure 4C).^22^^,^^23^ They had opposite relationships with structure and function, suggesting that keystone taxa mediated the divergent evolutionary trajectories in lake microbial communities over centennial scales. Universally negative correlation between annotated functional pathway proportions and keystone taxa revealed that increased keystone taxa abundance probably induced the emergence of unassigned function attributes, potentially reflecting enhanced functional novelty (Figure S4). In addition, keystone taxa usually had the ability to lead and interact with other taxa to maintain the microbial community network.^33^ The climatic shift from LIA to CWP induced an increase in the abundance of keystone taxa and strengthened interspecific and/or intraspecific cooperative interactions (Figure 4C), enabling more targeted resource utilization for survival (Figure S3). This resulted in a trade-off strategy between microbial community structure and function to ensure their persistence over centennial scales. Consequently, traditional genetic and metabolic processing was attenuated at the cellular level, allowing microorganisms to evolve novel functions to counter potential environmental changes (Figure S4). At the community level, this strategy resulted in a narrowed microbial niche breadth, characterized by decreased resource utilization and reduced genomic GC%, accompanied by a modest decline of microbial resistance to environmental disturbances (Figures 3B and 4C). These findings suggest that long-term microbial persistence under climate change may come at the cost of reduced ecological resilience and niche specialization.

### Microbial-driven biogeochemical processes under anthropogenetic climate change

Accompanying microbial structural and functional evolution, the biogeochemical cycling of nutrients in lakes has significantly modified in key processes and the intensity. Upon entering the CWP, human activities intensified, especially after 1950, greatly increasing atmospheric CO_2_ concentrations and consequently leading to rising global temperatures.^34^ Furthermore, anthropogenic nutrient loading into lakes accelerates eutrophication, resulting in microbial community restructuring and phytoplankton biomass accumulation.^35^^,^^36^

Stable carbon and nitrogen isotopic analyses serve as effective tools to trace these changes.^37^^,^^38^ In Lake Daihai, increased contribution of phytoplankton-derived carbon (C_phyto_) to the organic carbon pool will make δ^13^C_org_ more negative, as δ^13^C_phyto_ (the average of approximately −27‰)^24^^,^^37^ is usually lighter than δ^13^C of allochthonous soil organic matter (usually greater than −24‰ in Inner Mongolia).^39^^,^^40^ Sedimentary organic C/N ratios provide supporting evidence for this enhanced phytoplankton inputs (Figure 5A), as phytoplankton-derived organic matter typically exhibits lower C/N ratios (<10) compared to soil organic matter (>10).^37^^,^^41^ All of these resulted in a wider range of δ^13^C_org_ after 1950 (from −27.3 to −23.6‰) than the pre-1950 period (Figure 5A).

**Figure 5:**
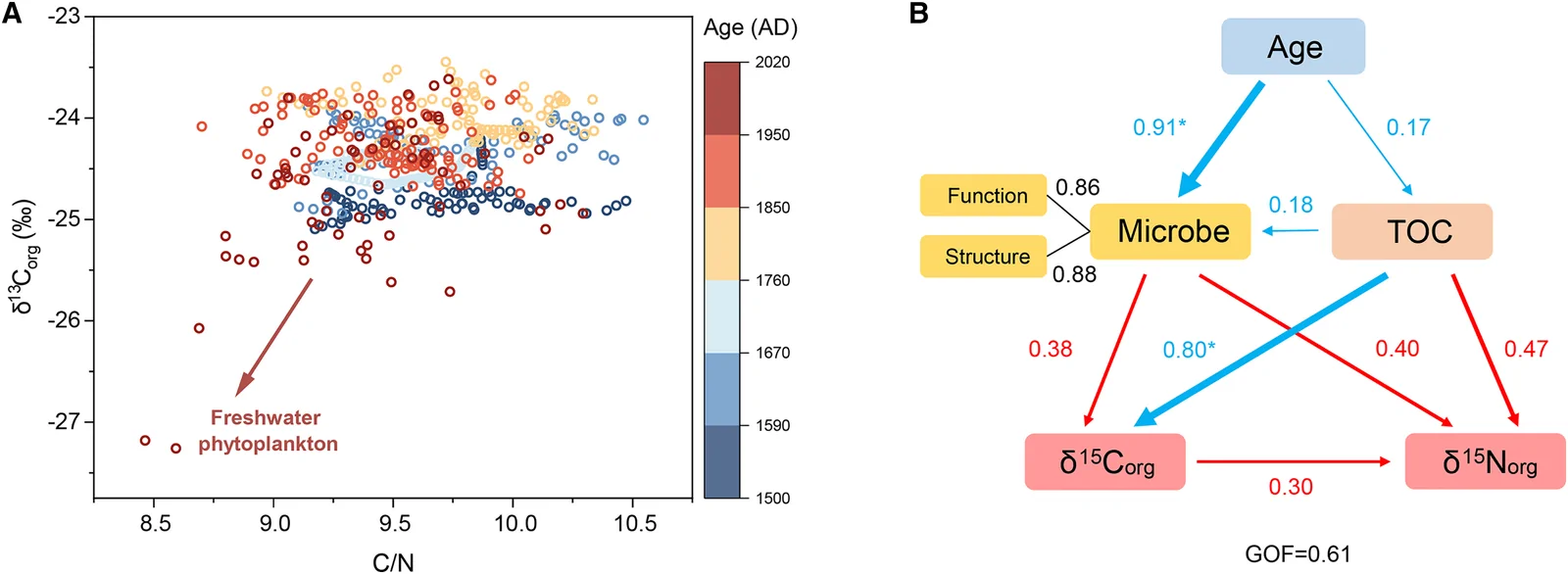
The relationship between sedimentary δ^13^C_org_ and C/N and partial least squares path model (A) Sedimentary δ^13^C_org_ versus C/N over the past 500 years in Lake Daihai. (B) Partial least squares path model quantifying direct relationships among key variables. The structure is characterized by the relative abundance of bacteria, while the function is characterized by the relative genomic GC content. Black lines and its labeled numbers represent the loadings between latent variables and observed variables. Red lines represent positive effects, blue lines represent negative effects. The numbers next to the arrows are path coefficients (R^2^), indicating the magnitude of the relationship’s impact. The significance level of each predictor is ∗, *p* < 0.05. The goodness of fit (GOF) is used to evaluate the model’s adequacy.

Therefore, with the structural evolution of the lake microbial community, δ^13^C_org_ showed corresponding changes, indicating altered carbon sources or biogeochemical processes (Figures 2C and 5B). Similarly, sedimentary δ^15^N_org_ exhibited dissimilarity over centennial scales, but it was significantly associated with the evolution of microbial function rather than community structure (Figure 2D). After 1950, anthropogenic nitrogen compounds (e.g., NO_3_^−^ and NH_4_^+^) with relatively low δ^15^*N* value were increasingly introduced into lakes and ultimately assimilated by microorganisms to synthesize proteins, which dominate the sedimentary organic nitrogen.^42^^,^^43^ The δ^15^*N* values of these proteins inherit the δ^15^N signature of the anthropogenic nitrogen source,^44^^,^^45^ leading to sedimentary δ^15^N_org_ values that are more negative than pre-1950 levels (Figure S1). Overall, the function and structure of microorganisms mediate the sedimentary δ^13^C_org_ and δ^15^N_org_ dynamics over a temporal series (Figure 5B).

It is worth noting that, compared with the apparent changes in microbial community structure and function observed after 1850, sedimentary δ^13^C_org_ and δ^15^N_org_ underwent obvious shifts nearly a century later (i.e., after 1950, Figures 1 and S1). This temporal lag likely occurs because these parameters represent different hierarchical levels of biological organization^46^^,^^47^: microbial community structure and function are assessed at the gene level, whereas δ^13^C_org_ and δ^15^N_org_ reflect ecosystem-level processes. Functional redundancy observed at the gene level allows microorganisms to maintain their core functions (such as metabolism) by buffering the impacts of species loss or succession under disturbances. For example, archaea can take over the ammonia-oxidation function of bacteria under certain extreme conditions.^48^ This gene-level redundancy could also buffer ecosystem-level metabolism (e.g., carbon mineralization and nitrogen fixation) across levels of biological organization, thereby stabilizing long-term biogeochemical functioning.^32^ As such, environmental information processing (e.g., chemotaxis, quorum sensing, and stress signal transduction) could be maintained by taxonomically distinct but functionally equivalent populations, decoupling community composition from ecosystem-level responsiveness.^49^ Those decoupling results in relatively stable ecosystem-level functions (i.e., δ^13^C_org_ and δ^15^N_org_) and explains their lagged responses. Consequently, the responses to climate change differ across biological hierarchical levels in lacustrine ecosystems.^50^ At the ecosystem level, in contrast to the simultaneous shifts in microbial communities and climate proxies (i.e., temperature and precipitation) after 1850, the sedimentary isotopic signal did not emerge until nearly a century later. This temporal decoupling implies that microorganisms are inherently sensitive to natural climate variability, whereas the delayed isotopic shift likely reflects the superimposed effects of both climatic and anthropogenic forcings, which required prolonged accumulation to be preserved in the sediment record.

This study investigated the microbial eco-evolutionary processes in Lake Daihai under the influence of the EASM over the past 500 years. The microbial community exhibited a significant temporal decay relationship, with deterministic processes dominating community assembly. We observed asynchronous divergence between microbial community structure and function, with subsequently organic δ^13^C and δ^15^N showing their respective evolution. The keystone taxa annotated as bacteria (rather than archaea, eukaryote, or virus) mediated the trade-off between microbial community structure and function in response to anthropogenic climate change. These findings, based on representative samples evaluated using multiple criteria, provided insights into microbial ecological strategies at the centennial scale.

### Limitations of the study

While our study provides insights into lake microbial community evolution under climatic and anthropogenic forcings, several limitations should be acknowledged. First, temporal resolution is constrained by the discrete nature of sediment sampling. Without continuous, high-resolution profiles, the precise timing of microbial shifts and the ability to distinguish abrupt disturbances from gradual trends remain difficult to resolve. Second, although our metagenomic sequencing depth was sufficient for dominant taxa, it may have under-sampled low-abundance species. This likely hampers the detection of rare taxa that may function as cryptic seed banks, leaving their ecological roles in long-term resilience largely unexplored. These constraints underscore the need for future studies with higher-resolution temporal sampling and deeper sequencing to more fully capture microbial-mediated responses to anthropogenic climate change.

## Resource availability

### Lead contact

Requests for further information and resources should be directed to and will be fulfilled by the lead contact, Wanzhu Li (liwanzhu@tju.edu.cn).

### Materials availability

This study did not generate new unique reagents.

### Data and code availability

• Raw metagenomic sequencing data were deposited in the Genome Sequence Archive (GSA) in Beijing Institute of Genomics (BIG) Data Center with accession number CRA019991.
• This paper does not report original code.
• The data reported in this paper have been deposited in Mendeley Data and are publicly available under the https://doi.org/10.17632/wgc5vc67jw.2.

## Acknowledgments

This study was jointly supported by the 10.13039/501100001809National Natural Science Foundation of China (grant number 42361144842 and 42361144879) and the Leading Talent Project of Guizhou Province (BQW[2025]001). The authors are grateful to the colleagues for their help in the field and the reviewers for their valuable comments.

## Author contributions

X.S.: writing – original draft, data curation, formal analysis, visualization. H.X.: writing – original draft, project administration, funding acquisition, conceptualization. W.L.: writing – original draft, data curation, formal analysis, visualization. J.W.: methodology, investigation, data curation. Y.S.: methodology, investigation. M.Y.: methodology, investigation. B.W.: writing – review and editing, conceptualization. C.-Q.L.: project administration, conceptualization.

## Declaration of interests

The authors declare no competing interests.

## STAR★Methods

### Key resources table

**Table:** 

REAGENT or RESOURCE	SOURCE	IDENTIFIER
**Deposited data**		

Raw metagenomic sequencing data	This paper; Beijing Institute of Genomics (BIG) Data Center	CRA019991
Source and analytical data	This paper; Mendeley Data	https://doi.org/10.17632/wgc5vc67jw.2

**Software and algorithms**		

R (version 4.4)	R Foundation for Statistical	RRID:SCR_001905
“ggplot2” package	Open access R package	RRID:SCR_014601
“iCAMP” package	Open access R package	RRID:SCR_028947
“reshape” packages	Open access R package	RRID:SCR_018983
“picante” package	Open access R package	RRID:SCR_028948
“spaa” package	Open access R package	RRID:SCR_028949
“VennDiagram” package	Open access R package	RRID:SCR_002414
“corrplot” package	Open access R package	RRID:SCR_024683
“ggClusterNet” package	Open access R package	RRID:SCR_028946
“Hmisc” package	Open access R package	RRID:SCR_022497
“plyr” package	Open access R package	RRID:SCR_026985
“vegan” package	Open access R package	RRID:SCR_011950
“AICcmodavg” package	Open access R package	RRID:SCR_028945
“car” packages	Open access R package	RRID:SCR_023299
“AICcmodavg” package	Open access R package	RRID:SCR_018551
MEGAHIT (v 1.1.3)	HKU-BGI	RRID:SCR_016368
Bowtie2 (v 2.2.4)	Bowtie2 software	RRID:SCR_009457
DIAMOND (v 0.8.23.85)	DIAMOND software	RRID:SCR_014601

### Method details

#### Study area and sampling

Lake Daihai (40°29′–40°37′N, 112°33′–112°46′E) is located in the transitional zone between the semi-arid and semi-humid regions in the temperate zone, influenced by the winter monsoon, westerlies, and East Asian Summer Monsoon (EASM). It represents a typical closed deep-water lake, with its water supply primarily recharged by surface runoff, groundwater inflow, and direct precipitation onto the lake surface.^35^ Historically, the Daihai Lake basin exhibited relatively weak anthropogenic disturbances, which gradually intensified around 1850 AD and accelerated markedly after AD 1950.^22^ Its unique climatic sensitivity and the evolutionary trajectory affected by anthropogenic influences make it a suitable site for investigating the evolution of lacustrine microbial communities and their responses to multiple driving factors. A 187-cm-long core (DH18-3) was collected from the central deep basin (40°35′06″N, 112°40′54″E) in August 2018 using a UWITEC gravity corer (60 mm diameter). The core was subsampled at 1-cm intervals, and seven sediment samples were selected for microbial analysis (Table S1).^51^ According to the DH18-3 age model established by ^137^Cs-^210^Pb and AMS ^14^C dating,^41^ these seven samples correspond to the Little Ice Age (approximately 1517–1850) and Current Warm Period (approximately 1850–2018) (Table S1). Details on the criteria for selecting sediment samples intended for microbial analysis were described in Methods S1. Total organic carbon to nitrogen ratios (C/N), stable carbon- and nitrogen-isotopic compositions (δ^13^C_org_ and δ^15^N_org_) of sedimentary samples were previously analyzed in Hu et al. (2023) and Wang et al. (2025).^27^^,^^35^ The C/N ratios were determined by an elemental analyzer (EA, Vario EL III, Germany), with an analytical precision of ± 0.1%. Sedimentary δ^13^C_org_ and δ^15^N_org_ were measured using a 253 Plus stable isotope ratio mass spectrometer (Thermo Fisher Scientific, USA), with an analytical precision of ± 0.2‰.

#### DNA extraction and metagenome sequencing

Total genomic DNA extraction was performed on seven sediment samples according to the manufacturer’s instructions of FastDNA SPIN Kit for Soil (MP Biomedical, USA). The DNA extracts were quantified with the dsDNA BR Assay kit (Invitrogen, USA) on a Qubit 3.0 Fluorometer (Life Technologies, CA, USA) following the manufacturer’s instructions. The quality of extracted DNA was checked with electrophoresis in a 1% agarose gel, and the sample purities were assessed using a NanoDrop Spectrophotometer. All DNA extracts were stored at −20°C prior to subsequent analyses. Shotgun metagenomic sequencing of seven sediment samples were conducted by Novogene Bioinformatics Technology Co., Ltd (Beijing, China). The clean reads were *de novo* assembled into scaffolds using MEGAHIT (version 1.1.3) and subsequently aligned to this gene catalog with Bowtie2 (version 2.2.4). The representative genes of each cluster with reads ≤2 were filtered out and the final metagenomic gene catalog (Unigenes) were obtained. Sequence data were aligned using DIAMOND (v0.8.23.85) software to assign the taxonomic profiles (i.e., bacteria, archaea, eukaryotes and virus). Details on the control standards for the metagenomic sequencing, metagenomic sequence assembly and gene catalog construction were referred to Methods S2. Raw metagenomic sequencing data were deposited in the Genome Sequence Archive (GSA) in Beijing Institute of Genomics (BIG) Data Center with accession number CRA019991. In this study, based on the previous published metagenomic data,^35^ Kyoto Encyclopedia of Genes and Genomes (KEGG) database was conducted to annotate the obtained Unigenes.

### Quantification and statistical analysis

All experimental data were analyzed using Origin 2025 and R software (version 4.5.1).^52^ For different figures, n represents the number of pairwise site differences (*n* = 21) or the number of sampling sites (*n* = 7). Linear regression was used to evaluate correlations between variables, with correlation coefficients (*r*) and *p*-values reported. For the relationship of temporal interval with the community structural similarity and functional pathway similarity (Figures 2A and 2B), the regression coefficients yielded *r* = -0.50 and *r* = -0.26. respectively. For the relationship between community structural dissimilarity and δ^13^C_org_ dissimilarity (Figure 2C), the regression coefficient yielded *r* = 0.58. For the relationship between functional pathway dissimilarity and δ^15^N_org_ dissimilarity (Figure 2D), the regression coefficient yielded *r* = -0.45. For the relationship between community structural similarity and functional pathway similarity (Figure 3A), the regression coefficients yielded *r* = 0.74 and *r* = 0.49 in LIA and LIA+CWP, respectively.

Comparisons between microbial community structural and functional characteristics were performed using the Mann-Whitney *U* test in LIA and CWP, with *p*-values reported. For the differences in Pos/Neg and PD between LIA and CWP (Figure 3B), *p*-values yielded 0.04 and 0.01, respectively. For the differences in GC and Niche between LIA and CWP (Figure 3B), *p*-values yielded 0.08 and 0.06, respectively. Spearman analysis was performed to quantify the relationships between keystone taxa and climatic and geochemical indexes (Figure 4C), with correlation coefficients (*r*) and *p*-values reported. The correlation coefficients varied from −0.96 to 0.96. Partial least squares path model was conducted to understand the direct and indirect relationships among parameters (Figure 5B), with path coefficients (R^2^), *p*-value and the goodness of fit (GOF) reported. The path coefficients ranged from 0.17 to 0.91, and the GOF was 0.61. All statistical significances were set at *p* < 0.05. Detailed information on statistical tests, sample sizes, and *p*-values was provided in the figure legends.

The “vegan” library was conducted to calculate the dissimilarity matrix^53^ to obtain the similarity and dissimilarity of microbial structural and functional composition and geochemical factors. The linear relationship between microbial community structural and functional similarity was established, and its slope <1 was used to as a threshold to occur the functional redundancy. The smoother the slope, the stronger the functional redundancy. For the microbial community assembly, “iCAMP” package was to quantify heterogeneous selection (Het), homogeneous selection (Hom), dispersal limitation (Dis), homogenizing dispersal (HomD), and drift and others (Dri); the first two as deterministic processes (Det) and the last three as stochastic processes (Sto).^54^ Cohesion calculated by the “reshape”, “Hmisc”, “plyr”, “vegan”, “AICcmodavg”, and “car” packages was used to quantify the microbial connectivity, with values greater than zero as a cooperative relationship (Pos) and less than zero as a competitive relationship (Neg). The Pos/Neg ratio was used to indicate the mode and intensity of microbial community connectivity.^55^^,^^56^ Phylogenetic diversity (PD), representing the sum of the branch lengths of microbial phylogenetic tree, was calculated using “picante” package.^57^ Niche width (Niche), reflecting the capability of their resource utilization,^58^^,^^59^ was calculated using “spaa” package. The “VennDiagram” package was used to build a Venn diagram.^60^ Spearman’s correlation analyses were performed using “corrplot” package.^61^ Partial least squares path model was conducted using the “plspm” package. Keystone taxa were identified by calculating the within-module connectivity (Zi) and between-module connectivity (Pi) for each node of microbial co-occurrence network using the “ggClusterNet” package.^62^ Different taxa represent their abundance having 2-fold difference at least between LIA and CWP.
