# Supplementary material for: Evolution of lake microbial structure and function under climatic and anthropogenic forcings: Insights from sedimentary ancient DNA
Source: iScience. 2026 Oct 3;29(10):117760. doi: 10.1016/j.isci.2026.117760 (PMC13661221; doi:10.1016/j.isci.2026.117760)
Supplement: Supplementary file 1 [file mmc1.pdf]

iScience, Volume *29*

Supplemental information

Evolution of lake microbial structure and function under climatic and anthropogenic forcings: Insights from sedimentary ancient DNA

Xinjie Shi, Hai Xu, Wanzhu Li, Jing Wang, Yunping Song, Meiling Yang, Baoli Wang, and Cong-Qiang Liu

Figure S1

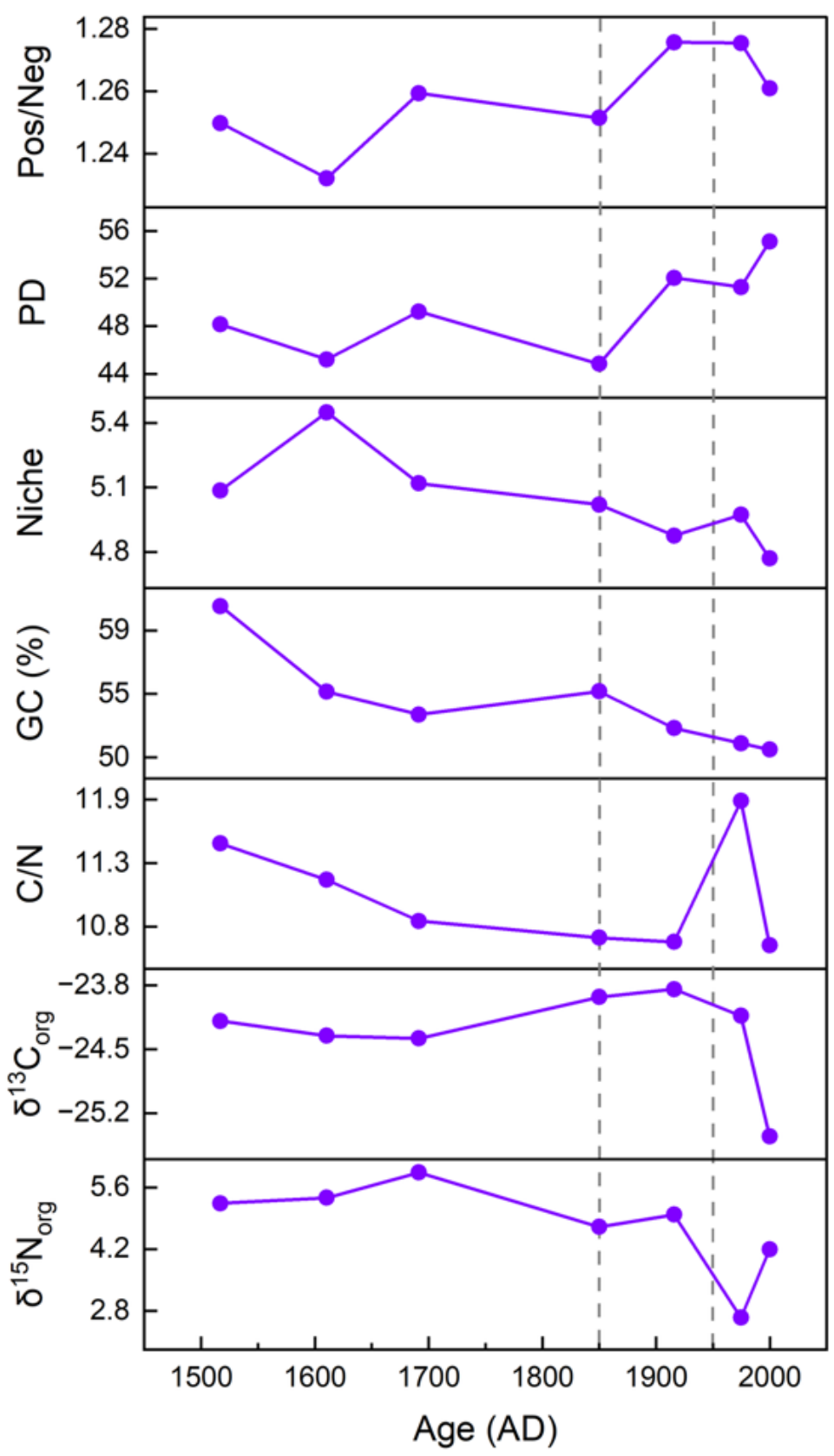

Figure S1. Variations in properties of microbial community structure and function and sedimentary $\delta^{13}C_{org}$ and $\delta^{15}N_{org}$. Pos/Neg, the ratio of cooperative to competitive relationship; PD, phylogenetic diversity; Niche, niche width; GC, microbial genome GC value; C/N, the ratio of total organic carbon to total nitrogen concentrations; $\delta^{13}C_{org}$, stable carbon isotopic composition of total organic carbon (‰); $\delta^{13}N_{org}$, stable nitrogen isotopic composition of total nitrogen (‰).

Figure S2

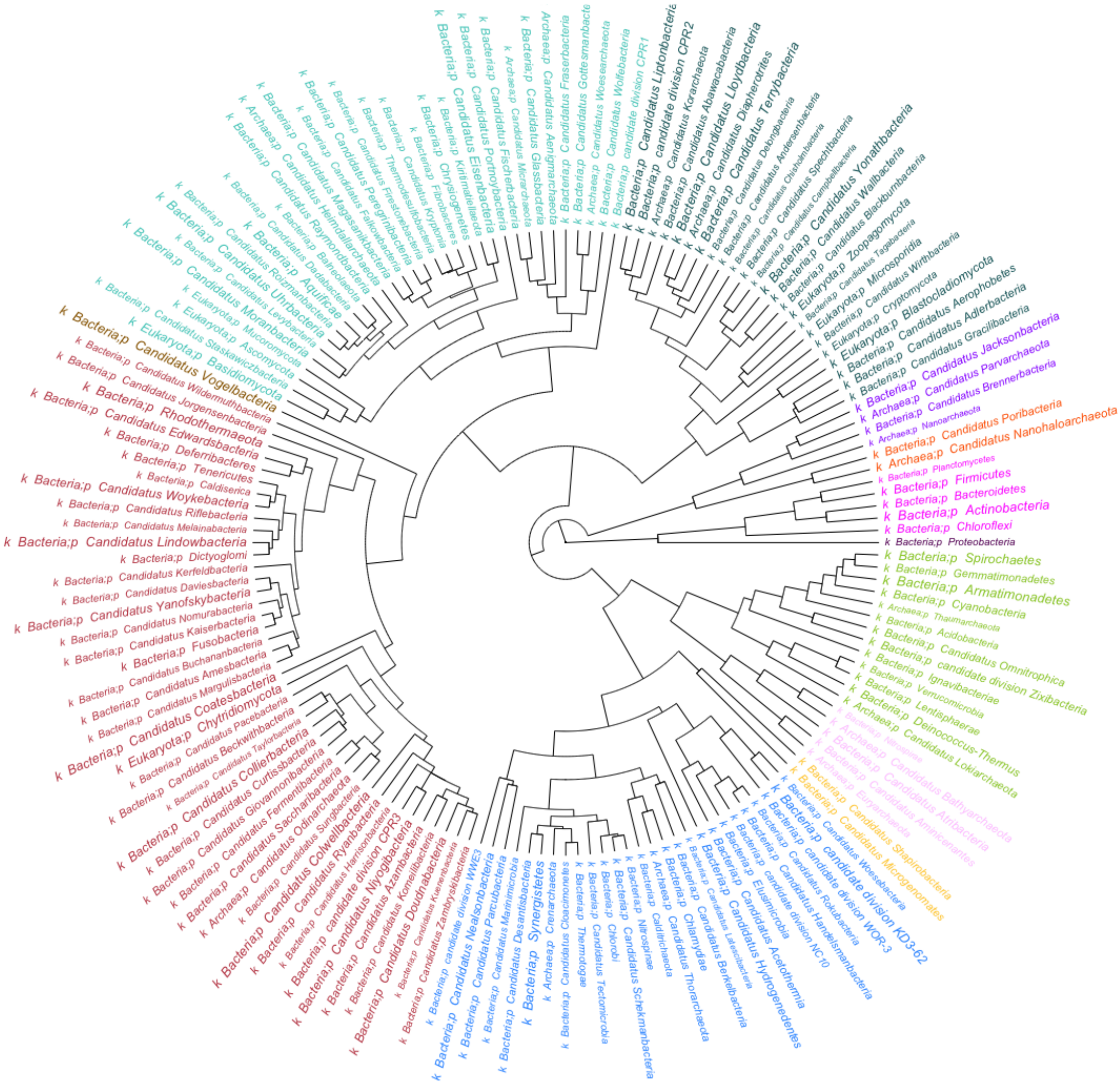

Figure S2. The phylogenetic tree of sedimentary microbial phyla. Different colors indicate diverse branches.

Figure S3

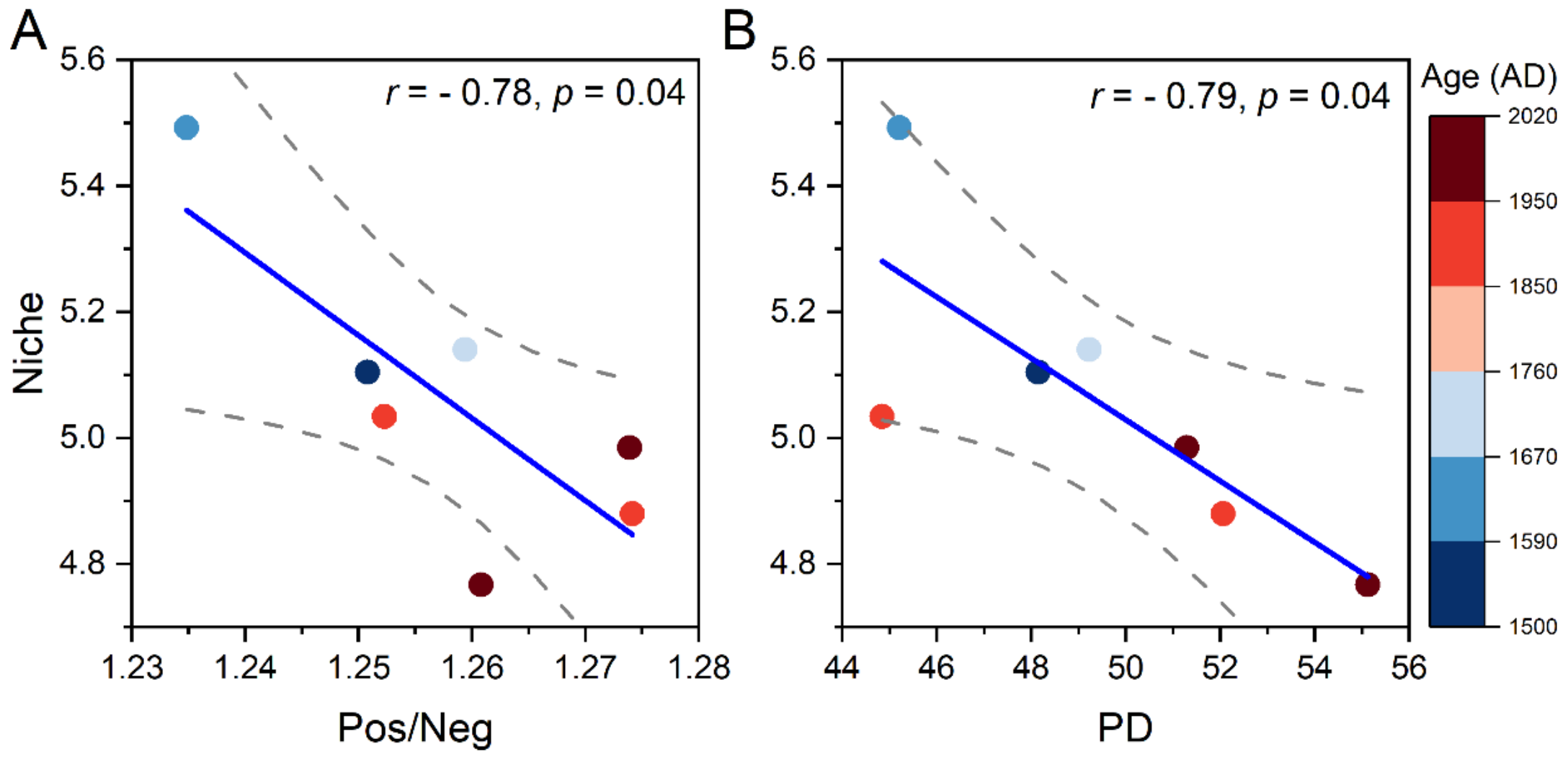

Figure S3. Relationships of cohesion, phylogenetic diversity and niche width. (A) Ratio of positive to negative cohesion (Pos/Neg) versus Niche width (Niche). **(B)** Phylogenetic diversity (PD) versus Niche width (Niche). n=7, n represents number of sites. Blue line is the fitting curve. The dotted lines indicate the 95% confidence intervals.

Figure S4

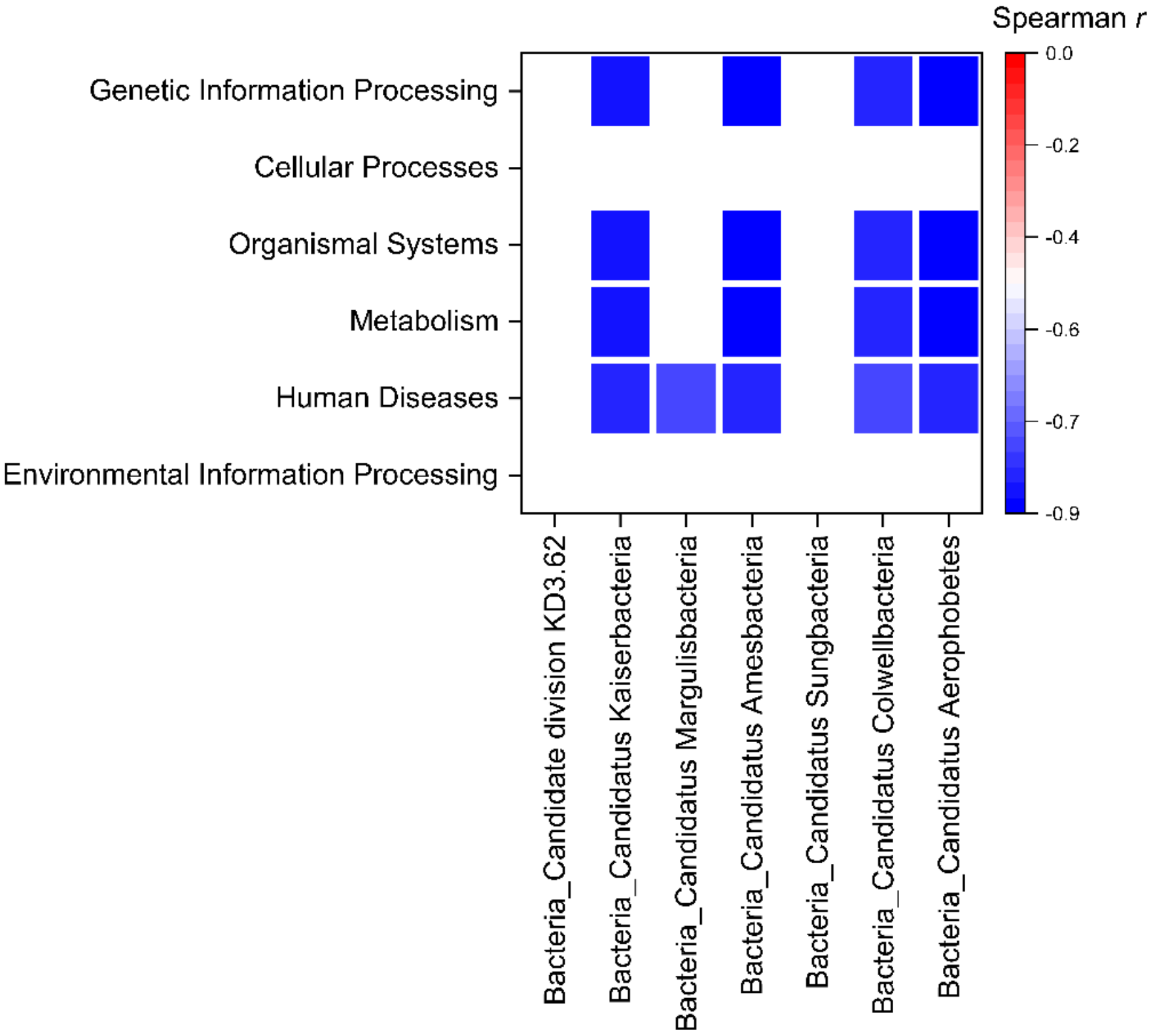

Figure S4. Spearman correlation plot between significantly different keystone taxa and functional pathways. n=7, n represents number of sites. The color block represents $p<0.05$.

Table S1. Sampling details of sedimentary core in Lake Daihai.

Layer	Age AD	Period
DH18-3-40	2000	Current Warm Period (CWP)
DH18-3-82	1975	Current Warm Period (CWP)
DH18-3-130	1916	Current Warm Period (CWP)
DH18-3-158	1850	Little Ice Age (LIA)
DH18-3-194	1692	Little Ice Age (LIA)
DH18-3-210	1611	Little Ice Age (LIA)
DH18-3-237	1517	Little Ice Age (LIA)

Methods S1. **The criteria for selecting sediment samples intended for microbial analysis.**

To ensure the scientific validity and representativeness of the microbial analysis, we selected seven sediment samples based on an established chronological framework. The selection criteria comprehensively considered the key climatic turning points, the changes in sedimentation rates, and the sensitivity of microbial communities to environmental disturbances. Specifically, these samples strategically cover the Little Ice Age (LIA) itself, the transition phase between the Little Ice Age (LIA) and the Current Warm Period (CWP) (~ 1850 AD), the early 20th-century warming period, and the period of significant human activity enhancement after 1950. In the sample selection process, we strictly adhered to the principle of balancing temporal continuity with representativeness: First, high-resolution dating data (^{137}Cs-^{210}Pb and ^{14}C dating method) were used to ensure each sample accurately corresponds to a specific climatic or environmental event horizon. Second, through sedimentation rate analysis, we selected the layers with stable deposition and no disturbance to avoid interference from sediment resuspension or bioturbation on microbial signals. Finally, sampling density was increased during a key climatic period (CWP), while sampling intervals were appropriately widened during a relatively stable climatic period (LIA). This approach ensures the integrity of the time series and simultaneously emphasizes the microbial response characteristics during key environmental periods, thereby enabling experimental samples to maintain temporal continuity and effectively capture ecological signals during abrupt climate change events.

Methods S2. **Details on the control standards for the metagenomic sequencing, metagenomic sequence assembly and gene catalogue construction.**

To ensure the reliability and reproducibility of the metagenomic sequencing data, we implemented rigorous quality control standards during data preprocessing. Raw sequencing reads were first processed to remove adapter contamination using Cutadapt (v2.6), allowing a maximum mismatch rate of 10% in the adapter sequence. [1] For low-quality read filtering, we employed Trimmomatic (v0.39) with a sliding window of 4 bases,[2] trimming reads when the average quality score dropped below Q15, and subsequently discarding any read with a length less than 75% of the original read length or shorter than 50 bp. Reads containing ambiguous bases (Ns) exceeding 5% of the read length were also eliminated. To address duplicate reads, which may arise from PCR amplification bias during library preparation, we applied FastUniq to identify and remove identical duplicate reads,[3] retaining only unique sequences for downstream analysis. The resulting clean reads were assessed using FastQC to ensure high data quality, with the following criteria [4]: (1) the per-base sequence quality (Q20 and Q30) exceeding 95% and 90%, respectively; (2) no remaining adapter contamination or overrepresented sequences; and (3) a balanced base composition without significant GC bias. These stringent quality control measures guarantee the integrity of the dataset and provide a solid foundation for the reproducibility of subsequent taxonomic and functional analyses.

The post-QC clean reads (with an average of 6.21 Gb per sample) were subsequently de novo assembled into scaftigs using MEGAHIT (version 1.1.3) [5] with the following parameters: -end-to-end, -sensitive, -I 200, -X 400. Open reading frames (ORFs) were predicted from scaftigs (≥500 bp) for each sample using MetaGeneMark (option gene_prediction) (version 2.10).[6] From the prediction results, only genes with minimum ORF length greater than or equal to 100 nt were retained via the parameter -l 100. All predicted genes across the 7 sediment samples were combined together and a non-redundant initial gene catalogue was clustered using CD-HIT (version 4.8.1) [7]

with a 95 % sequence identity threshold using the following settings: -c 0.95, -G 0, -aS 0.9, -g 1, -d 0. Clean reads from each sample were then aligned to this gene catalogue with Bowtie2 (version 2.2.4) [8] using the following parameters: -end-to-end, -sensitive, -I 200, -X 400. The representative genes of each cluster with reads ≤ 2 were filtered out and the final metagenomic gene catalogues (Unigenes) of the Lake Daihai microbiome were obtained.

Reference

1. Martin, M. (2011). Cutadapt removes adapter sequences from high-throughput sequencing reads. EMBnet.journal. 17, 10-12. https://doi.org/10.14806/ej.17.1.200.
2. Bolger, A.M., Lohse, M., and Usadel, B. (2014). Trimmomatic: a flexible trimmer for Illumina sequence data. Bioinformatics. 30, 2114-2120. https://doi.org/10.1093/bioinformatics/btu170.
3. Xu, H., Luo, X., Qian, J., Pang, X., Song, J., Qian, G., Chen, J., and Chen, S. (2012). FastUniq: a fast de novo duplicates removal tool for paired short reads. PLoS ONE. 7, e52249. https://doi.org/10.1371/journal.pone.0052249.
4. de Sena Brandine, G., and Smith, A.D. (2019). Falco: high-speed FastQC emulation for quality control of sequencing data. F1000Research. 8, 1874. https://doi.org/10.12688/f1000research.21142.2.
5. Li, D., Luo, R., Liu, C.M., Leung, C.M., Ting, H.F., Sadakane, K., Yamashita, H., and Lam, T.W. (2016). MEGAHIT v1.0: A fast and scalable metagenome assembler driven by advanced methodologies and community practices. Methods. 102, 3-11. https://doi.org/10.1016/j.ymeth.2016.02.020.
6. Zhu, W., Lomsadze, A., and Borodovsky, M. (2010). Ab initio gene identification in metagenomic sequences. Nucleic Acids Res. 38, e132. https://doi.org/10.1093/nar/gkq275.
7. Fu, L., Niu, B., Zhu, Z., Wu, S., and Li, W. (2012). CD-HIT: accelerated for clustering the next-generation sequencing data. Bioinformatics. 28, 3150-3152. https://doi.org/10.1093/bioinformatics/bts565.
8. Langmead, B., and Salzberg, S.L. (2012). Fast gapped-read alignment with Bowtie 2. Nat. Meth. 9, 357-359. https://doi.org/10.1038/nmeth.1923.
